# Some Thoughts on the Care and Management of the Deciduous Teeth

**Published:** 1897-02

**Authors:** S. H. Guilford


### SOME THOUGHTS ON THE CARE AND MANAGEMENT OF THE DECIDUOUS TEETH.[1]

BY S. H. GUILFORD, D.D.S., PH.D.

Mr. President and Fellow-Members,—Wordsworth has beautifully said in his poetic way, "The child is father to the man." If this be true, and it doubtless is, that the character, temperament, and possibilities of the future man are shadowed forth in the earlier years of child-life, and that conversely the tendencies and proclivities, as well as the training, of childhood will affect and determine the character of the future man, may we not justly

[1] Read before the Academy of Stomatology, Philadelphia, December 22, 1896.

conclude that in a more material way the physical condition of the child will, to a great extent, determine the well-being of the man?

Weakly children do not usually develop into strong adults, nor vigorous children into delicate men. Of the various organs upon whose normal functional activities the health of the adult depends, none, perhaps, are more important than the organs of mastication, which guard the entrance to one of the most important of all organs,—the stomach.

In health the importance of the teeth is not apt to be fully appreciated, but in their absence or with their activities impaired, the resultant ills not only emphasize their usefulness, but often do it in a painful and health-destroying way.

The health of the individual, however, so far as the teeth are responsible, is not alone dependent upon or influenced by those of the permanent set, for the deciduous teeth play an earlier and most important part in determining the future well-being of the individual.

With mastication imperfectly or indifferently performed in childhood, the stomach often becomes so weakened in functional power by over-taxation as to never fully recover its normal condition. So, also, the suffering that the child is subjected to through decayed and tender dental organs will often produce a permanently deleterious effect upon the delicate nervous organism, and modify, if not entirely change, the natural disposition of the individual.

Less serious, though perhaps scarcely less important, is the humanitarian side of the question, which leads us to regard the comfort of the child as well as its health.

In view of these facts, the care and preservation of the deciduous teeth become an all-important matter to the child and a subject worthy of most careful consideration on the part of ourselves as practitioners.

As to the care of these organs, two essential points present themselves: one is that of instructing the parent how to properly brush and cleanse them, and the other that of impressing the importance of regular periodical visits ot the child to the dentist for the examination of the teeth and receiving advice concerning them.

The cleansing of the teeth of young children should be done with a small and soft brush, and performed twice daily, morning and evening, by the mother or nurse. It should be done regularly, carefully, and conscientiously; and while the child may not understand the object of it, a habit will gradually be formed which will lead to its continuance in after-years.

The visits to the dentist should not be less frequent than four times a year, upon which occasions most careful examinations should be made for caries, all discoloration or stain removed, and the indications of the eruption of the permanent teeth watched for.

These regular visits, unattended by pain, discomfort, or fatigue, will not only accustom the child to the surroundings of the dental office and forestall any natural dread, but they will lead to the establishment of relations of intimacy and confidence between the practitioner and his little patient, which will make future and less agreeable operations more easy of accomplishment.

When incipient decay is noted, either upon the exposed or approximal surfaces of the teeth, it must at once be removed, and when minute cavities are discovered they will have to be filled. The frequent visits will lead to the early discovery of decay and its easy remedy.

So, too, when the time arrives for the shedding of the teeth, the practitioner can notice their gradual loosening and at the proper time remove them.

With the confidence of the child once secured, the extraction of a loose deciduous tooth can easily be accomplished.

In this way, by means of the regular visits of the patient and the careful scrutiny and faithful service of the practitioner, the child, if of average health, should be enabled to pass through the period of early childhood without suffering or discomfort, and reach the time of youth with the dental organs in the best possible condition.

Unfortunately for us, however, and for our patients as well, no such Utopian conditions exist, and we have to accept things as they are and not as we would have them. Whilst we can see the little members of our regular families periodically, and carry along their dental organs in the easy and comfortable manner just outlined, in too many cases they are only presented at long and irregular intervals, after caries has made serious inroads and the organs are more or less permanently injured.

This makes our task more difficult and involves a more trying ordeal for the patient.

The one operation most frequently called for in the deciduous as well as the permanent teeth is filling, and the question at once arises as to how we may perform the operation with the minimum of pain and discomfort and the maximum of usefulness.

Owing to existing conditions the procedure must vary considerably from that followed in operating upon the permanent teeth, both in regard to details and the character of materials employed.

Gold, the mainstay of the operator for the permanent teeth, is virtually interdicted for the deciduous ones, on account of the difficulties of its introduction, and we are, therefore, forced to depend upon materials of a plastic nature. Fortunately, however, these materials, though of such limited durability, can, in the hands of the skilful, be made to serve the necessary purposes during the comparatively short period that these organs remain in active service.

Of the plastics most commonly used, zinc phosphate is the least valuable, on account of its slight durability and the danger of pulp devitalization, which usually attends its employment.

Being easy of preparation and introduction, its use is far too common, although occasionally it serves a valuable purpose in large cavities with frail walls, where other materials would be most difficult of introduction and retention.

Amalgam is not a sightly substance when placed in contrast with fair tooth substance, but its durability, together with its rapid and easy introduction, renders it of greatest service to us in the posterior teeth, where its inharmony of color will not be apparent, but its employment in the anterior teeth is uncalled for and totally unjustifiable.

For the incisors and cuspids we have in gutta-percha a material without a peer. Although more difficult of introduction than the other plastics referred to, it is far more durable than zinc phosphate, is non-irritant, non-conductive, and easily repaired or added to.

For many years your essayist has made it an almost invariable rule to employ amalgam for the posterior deciduous teeth and gutta-percha for the anterior teeth.

Complications are frequently encountered, however, in our endeavors to save these teeth that tax our skill and ingenuity to the utmost. After excavation the pulp is often found to be nearly or quite exposed, and in other instances it has become devitalized and abscess has supervened. How to treat these conditions to the best advantage of the patient often presents a serious problem.

If it be important to preserve the vitality of a pulp in the permanent teeth, as most of us will agree, it is even more so in the deciduous set; for while in the one case it is no difficult matter to devitalize and remove a pulp and successfully treat and fill the root-canal, in the deciduous teeth the operation is beset with many difficulties and the after good results are not by any means as certain.

Besides this, we must remember that while a healthy devitalized permanent tooth will be serviceable and not interfere with nature's processes, a deciduous tooth similarly conditioned often materially obstructs the physiological changes incident to second dentition, for in most cases after the death of the pulp the normal resorption of the root ceases. This might prove of minor importance were it not that the condition seriously impedes the eruption of the permanent successor, often causing it to erupt out of its normal position and producing irregularity.

With these facts in mind, it behooves us to employ our best skill to preserve the life of the pulp in every case where it is possible. This can in most instances be done by the usual method of capping, and while the operation is attended with difficulties not met with to the same degree in the permanent teeth, it is always worthy of our best efforts.

Where the devitalization of the pulp is unavoidable, it should afterwards be removed with the greatest care and the root-canal treated antiseptically and filled.

In my own practice I obtain very satisfactory results by packing the canal as tightly as possible with cotton and iodoform paste. The method pursued by some, of allowing the dead pulp to remain and then drilling a vent below the gum line for the escape of the gaseous products of decomposition, is unscientific in character and disgusting in results; whereas the other practice of removing the pulp and leaving the canal unfilled is simply to invite future trouble, with small chance of being disappointed.

In the treatment of abscess at the roots of the deciduous teeth we meet with our greatest difficulty, on account of the tender years of the patient, the large apical foramen, and the uncertainty of the result. However, if the patient be tractable, it is best to put forth the effort to cure, and, if unsuccessful, we have at least the last resort of filling the root antiseptically, and thus lessening, if not remedying, the disease. Where the tooth thus affected is approaching the period of displacement and the condition is not attended with discomfort, we may be justified in non-interference.

In extreme cases of suffering, where relief cannot be obtained in any other way, extraction may be resorted to; but it should in all cases be a last resort, for the removal of any of the deciduous teeth before the proper time will frequently lay the foundation of irregularity in the permanent set.

One point remains to be touched upon, and that is the form in which the approximal surfaces should be left after filling. A few practitioners believe that the contour should be restored as in the permanent teeth and for the same reasons; whereas others, and the majority, practise permanent separation, for the reason that it simplifies the operation and is likely to work no harm in the short time that these teeth are retained.

I believe the latter plan the wiser and better one, all things considered, but in either case it appears reasonable that a permanent separation should be made between the deciduous second and the permanent first molar where the former has become decayed upon its distal surface. In almost all such cases the mesial surface of the permanent tooth is found to have been injured by caries, and its extension can best be checked by the prevention of subsequent contact.

